# Inositol pentakisphosphate isomers bind PH domains with varying specificity and inhibit phosphoinositide interactions

**DOI:** 10.1186/1472-6807-11-11

**Published:** 2011-02-10

**Authors:** Sean G Jackson, Sarra Al-Saigh, Carsten Schultz, Murray S Junop

**Affiliations:** 1 Department of Biochemistry and Biomedical Sciences, McMaster University, 1200 Main Street West, Hamilton, ON, L8N 3Z5, Canada; 2 Cell Biology and Cell Biophysics Unit, European Molecular Biology Laboratory, Heidelberg, Germany

## Abstract

**Background:**

PH domains represent one of the most common domains in the human proteome. These domains are recognized as important mediators of protein-phosphoinositide and protein-protein interactions. Phosphoinositides are lipid components of the membrane that function as signaling molecules by targeting proteins to their sites of action. Phosphoinositide based signaling pathways govern a diverse range of important cellular processes including membrane remodeling, differentiation, proliferation and survival. *Myo-*Inositol phosphates are soluble signaling molecules that are structurally similar to the head groups of phosphoinositides. These molecules have been proposed to function, at least in part, by regulating PH domain-phosphoinositide interactions. Given the structural similarity of inositol phosphates we were interested in examining the specificity of PH domains towards the family of *myo-*inositol pentakisphosphate isomers.

**Results:**

In work reported here we demonstrate that the C-terminal PH domain of pleckstrin possesses the specificity required to discriminate between different *myo-*inositol pentakisphosphate isomers. The structural basis for this specificity was determined using high-resolution crystal structures. Moreover, we show that while the PH domain of Grp1 does not possess this high degree of specificity, the PH domain of protein kinase B does.

**Conclusions:**

These results demonstrate that some PH domains possess enough specificity to discriminate between myo-inositol pentakisphosphate isomers allowing for these molecules to differentially regulate interactions with phosphoinositides. Furthermore, this work contributes to the growing body of evidence supporting *myo*-inositol phosphates as regulators of important PH domain-phosphoinositide interactions. Finally, in addition to expanding our knowledge of cellular signaling, these results provide a basis for developing tools to probe biological pathways.

## Background

**Figure 1:**
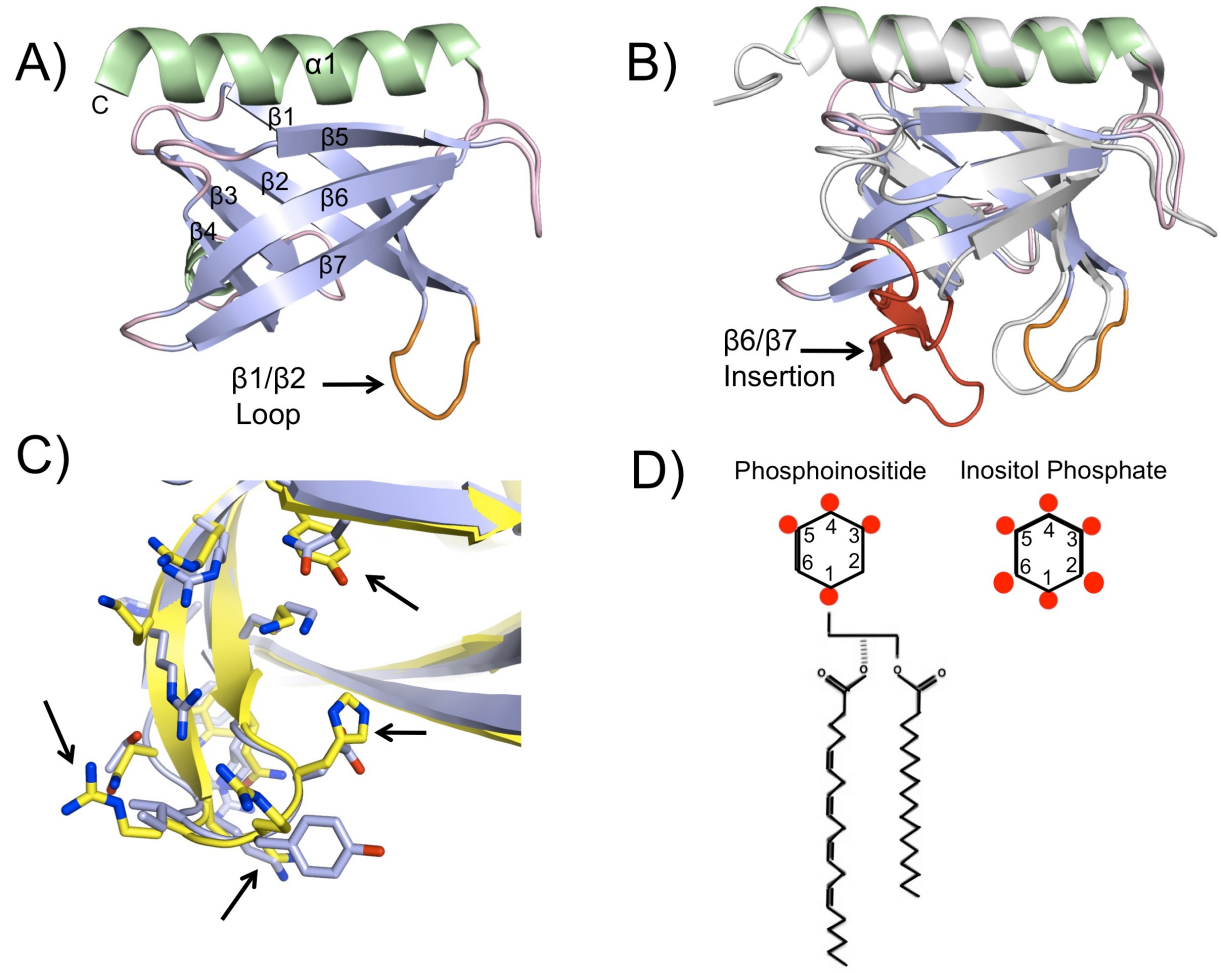
**PH domains and their ligands**. Schematic representation of a typical PH domain is shown in panel A (Akt PH domain, residues 111-115 omitted, PDB code 1UNQ). The core β sandwich is colored in blue, C-terminal α helix in green, loops in light pink and the β1/β2 loop in orange. In panel B the PH domains from Grp1 (PDB code: 1FGY) and Akt (PDB code: 1UNQ) are aligned to highlight a unique structural feature (colored in red) of the Grp1 PH domain (shown in grey). The variable β1/β2 loops of CPH (PDB code: 1ZM0) and Akt (PDB code: 1UNQ) are illustrated in panel C and colored yellow and blue respectively. Black arrows highlight key differences in binding pockets. Panel D illustrates the differences between phosphoinositides and inositol phosphates. Phosphate groups are shown as red dots.

PH (pleckstrin homology) domains represent one of the most widely distributed domains in the human proteome, being found in over 250 human proteins [1] involved in a wide range of diverse biological function (reviewed in [2]). Despite limited sequence similarity, PH domains maintain a highly conserved architecture (Figure 1, panel A) consisting of a seven-stranded anti-parallel β sandwich closed at one end by a C-terminal α helix. The opposing end remains open and accommodates several variable loops. Loop β1/β2, located between the first and second β strands often contains positively charged residues involved in ligand binding. In some PH domains this binding pocket region is extended, adopting additional secondary structure elements (Figure 1, panel B). Ligand specificity is therefore determined primarily by the overall structure and composition of the binding pocket region [3,4].

Soon after their discovery, PH domains were shown to be important for targeting host proteins to specific sites at the membrane via specific interactions with various phosphoinositides [5,6]. Phosphoinositides are lipid components of the membrane that act as key signaling molecules [7]. These lipids contain an inositol head group that can be reversibly phosphorylated at the 3, 4 and 5 positions to yield seven different phosphoinositides (Figure 1, panel D). Phosphoinositides propagate cellular signals by recruiting specific signaling proteins to the membrane by means of phosphorylated inositol head groups. In addition to comprising the head groups of phosphoinositides, soluble *myo-*inositol phosphates (IPs) themselves also function as important second messengers [8,9]. In contrast to phosphoinositides, *myo-*inositol phosphates are reversibly phosphorylated at any or all of the six positions about the inositol ring, giving rise to a large variety of different signaling molecules. Phosphate groups attached to the inositol ring adopt equatorial positions at 5 of the 6 possible locations. The remaining position is axial. This distribution of 5 equitorial and 1 axial positions is a key feature of myo-inositol phosphates. Depending on the degree of phosphorylation and the position of the phosphates, the inositol ring itself adopts different conformations further adding to the stereospecific diversity of this class of signaling molecules.

*myo-*Inositol phosphates such as IP_3 _have been very well characterized and shown to act as second messengers by directly binding target proteins and modulating activity [10-12]. In principle, the very abundant IP varieties could also act as signaling molecules by directly regulating interactions of PH domains with their phosphoinositide ligands [10,13,14]. This is a particularly attractive hypothesis given the structural diversity of inositol phosphates. Diversity in inositol phosphate structure could result in interactions with PH domains that vary in terms of both mode of binding and affinity. If so, IPs would provide cells with a mechanism for fine tuning the many important PH domain-phosphoinositide signaling interactions. Experimental evidence in support of *myo-*inositol phosphates regulating PH domain-phosphoinositide interactions is growing rapidly. IP_3 _was shown to efficiently dissociate the phospholipase C (PLC) PH domain/PtdIns(4,5)P_2 _interaction releasing PLC from the membrane when added exogenously or generated in cells [13,15]. Perhaps the most notable example however, of an *myo-*inositol phosphate regulating PH domain binding is that of the protein kinase B (Akt) signaling pathway. Here, IP_5(2)_, (nomenclature for myo-inositol pentaphosphates adopted here is as follows; IP_5(#)_, *myo*-inositol pentaphosphate, number in brackets indicates position missing a phosphate) one of the most abundant inositol phosphates in most cell, was shown to compete with PtdIns(3,4,5)P_3 _for binding to the PH domain of Akt (PH_Akt_) thereby preventing its membrane localization and subsequent activation [14].

Pleckstrin's carboxyl terminal PH domain (CPH) is known to bind PtdIns(3,4)P_2 _[16]. In a previous report, we showed that the *myo-*inositol pentaphosphate (IP_5_), IP_5(4)_, is a particularly effective inhibitor of CPH binding to PtdIns(3,4)P_2 _[17]. Given the central role of PH domains in cell signaling and the large diversity of IPs, we were interested in further analyzing the specificity of binding for all IP_5 _isomers to several different PH domains. The present study examines whether CPH is capable of specifically recognizing different IP_5 _isomers. Using high resolution crystal structures the structural basis for observed IP_5 _specificity is further examined. Finally, we extend the study to include 2 additional PH domains from Grp1 and Akt. This work demonstrates that CPH possesses the specificity required to differentiate between different IP_5 _isomers. This high degree of specificity was also found to be a property of the PH_Akt _but not the Grp1 PH domain (PH_Grp1_). Together, results presented here support a role for *myo-*inositol pentakisphosphates as regulators of PH domain/phosphoinositide signaling and further demonstrate their potential use as potent inhibitors of cell signaling.

## Results and Discussion

### Inhibition of CPH/PtdIns(3,4)P_2 _binding by inositol pentakisphosphates

**Figure 2:**
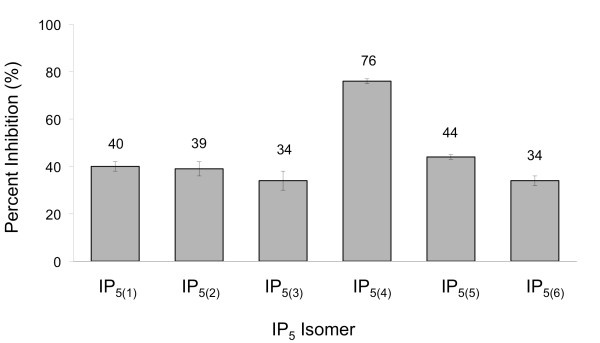
**Inhibition of CPH binding**. Inhibition of CPH (50 µM) binding to liposomes containing PtdIns(3,4)P_2 _by *myo*-inositol pentakisphosphates (100 µM). Percent inhibition is plotted on the y-axis for the different IP_5 _isomers. Percent inhibition values are shown above the bars. Error bars represent +/- standard error of mean (SEM).

A previous study suggested that the inositol pentakisphosphate, IP_5(4)_, is a particularly effective inhibitor of CPH binding to PtdIns(3,4)P_2 _[17]. Since there are six different IP_5_'s we sought to determine whether individual IP_5 _isomers differed in their ability to inhibit CPH/phosphoinositide interactions. This is an interesting question since IP_5 _isomers are structurally quite similar, varying only in the position of the 5 phosphate groups about their 6-carbon ring. A difference in inhibitory properties would suggest that CPH possesses a high degree of binding specificity enabling it to discriminate between very subtle differences in ligands. The ability of IP_5_'s to disrupt CPH/phosphoinositide interactions was determined using SPR. In this assay, liposomes containing PtdIns(3,4)P_2 _were immobilized to the sensor surface and CPH binding determined in the presence and absence of each IP_5 _isomer. As shown in Figure 2, the family of IP_5 _isomers does in fact differ in their inhibitory properties (P value < 0.0001). IP_5(4) _was a significantly better binder relative to other IP_5 _molecules. The remaining IP_5 _isomers (IP_5(1)_, IP_5(2)_, IP_5(3)_, IP_5(5) _and IP_5(6)_) all had similar affinities for CPH. The weakest inhibitor of CPH binding was IP_5(3)_. These observed differences in affinity indicate that the binding pocket region of CPH is able to differentiate between various arrangements of phosphate groups in the IP_5 _family. The arrangement of phosphates displayed by IP_5(4) _permits a particularly effective mode for interaction with CPH (76% inhibition), presumably by accommodating a favorable interaction not possible for other IP_5 _isomers. In a previous study our group determined the crystal structure of CPH bound to IP_5(4)_. To elucidate the structural basis for the observed specificity we determined the crystal structure of CPH bound to one of the lower affinity isomers (IP_5(6)_) thereby allowing for a detailed structural comparison.

### Structure of CPH/IP_5(6) _complex

**Figure 3:**
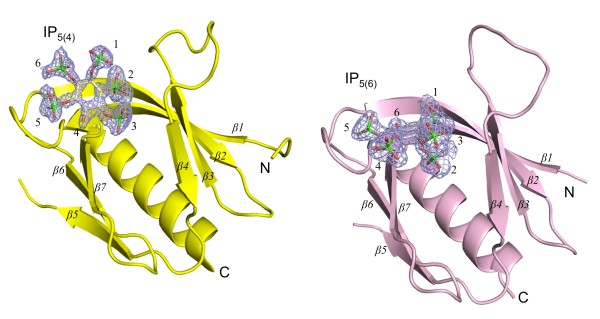
**CPH/IP_5 _structures**. Cartoon representations of the crystal structures of CPH bound to IP_5(4) _and IP_5(6)_. The CPH/IP_5(4) _structure is shown in yellow and the CPH/IP_5(6) _structure in pink. Electron density maps (2fo-fc) have been contoured at a sigma level of 2.0.

The crystal structure of CPH bound to IP_5(6) _was determined to 1.7 Å and solved by molecular replacement using the apo-CPH structure (PDB code 1ZM0) as a search model. Data collection and model refinement statistics are shown in Table 1. The final model, refined to R and R_free _values of 0.18 and 0.24, was well ordered with the exception of the β5/β6 loop (residues 301-310). This loop was similarly disordered in previous structures of CPH suggesting it is inherently flexible and most likely not directly involved in ligand binding [17,18]. Three monomers of CPH were present in the asymmetric unit. Each of these monomers was highly similar to the others, having shared r.m.s deviations of less than 0.2 Å. This similarity extended to the bound IP5 molecules which were all refined with full occupancy in the final model. The presence of IP5 resulted in only minor crystal contacts between monomers (IP5_A_-R257_C_; IP5_B_-R257_B'_; IP5_C_-R257_A_) and its mode of binding to the CPH domain is therefore not expected to be influenced significantly by these crystal contacts. Comparison of the CPH/IP_5(6) _structure with the CPH/IP_5(4) _structure revealed no significant conformational changes in the overall structure with one exception being the β3/β4 loop which adopted variable conformations as shown in Figure 3.

**Table 1:** Crystallographic and data refinement statistics.

	CPH/IP_5(6)_
**Date Collection**	
**Space group**	C2
**Unit-cell parameters (Å)**	a = 82.5, b = 47.6 and c = 87.6 α = β = γ = 90
**No. of molecules in asymmetric unit**	3
**Resolution range (Å)^a^**	50.00 - 1.65 (1.71-1.65)
**Unique reflections**	39 679
**Data Redundancy^a^**	3.5 (2.5)
**Completeness (%)^a^**	98.47 (91.4)
**I/σ(I)^a^**	26.6 (2.4)
**R_merge _(%)^a^**	3.9 (36.3)
**Model and refinement**	
**Resolution range (Å)^a^**	87.71-1.75 (1.79-1.75)
**R_work _(%)**	18.0
**R_free _(%)**	24.3
**No. of reflections**	31 402 (28 793 in working set and 2609 in test set)
**No. of waters**	315
**r.m.s.d bond lengths (Å)**	0.024
**r.m.s.d bond angles (°)**	2.3
**Average *B *factor (Å^2^)**	40.1
**PDB code**	2I5C

^a^Data for the highest resolution shell are shown in parentheses.

**Figure 4:**
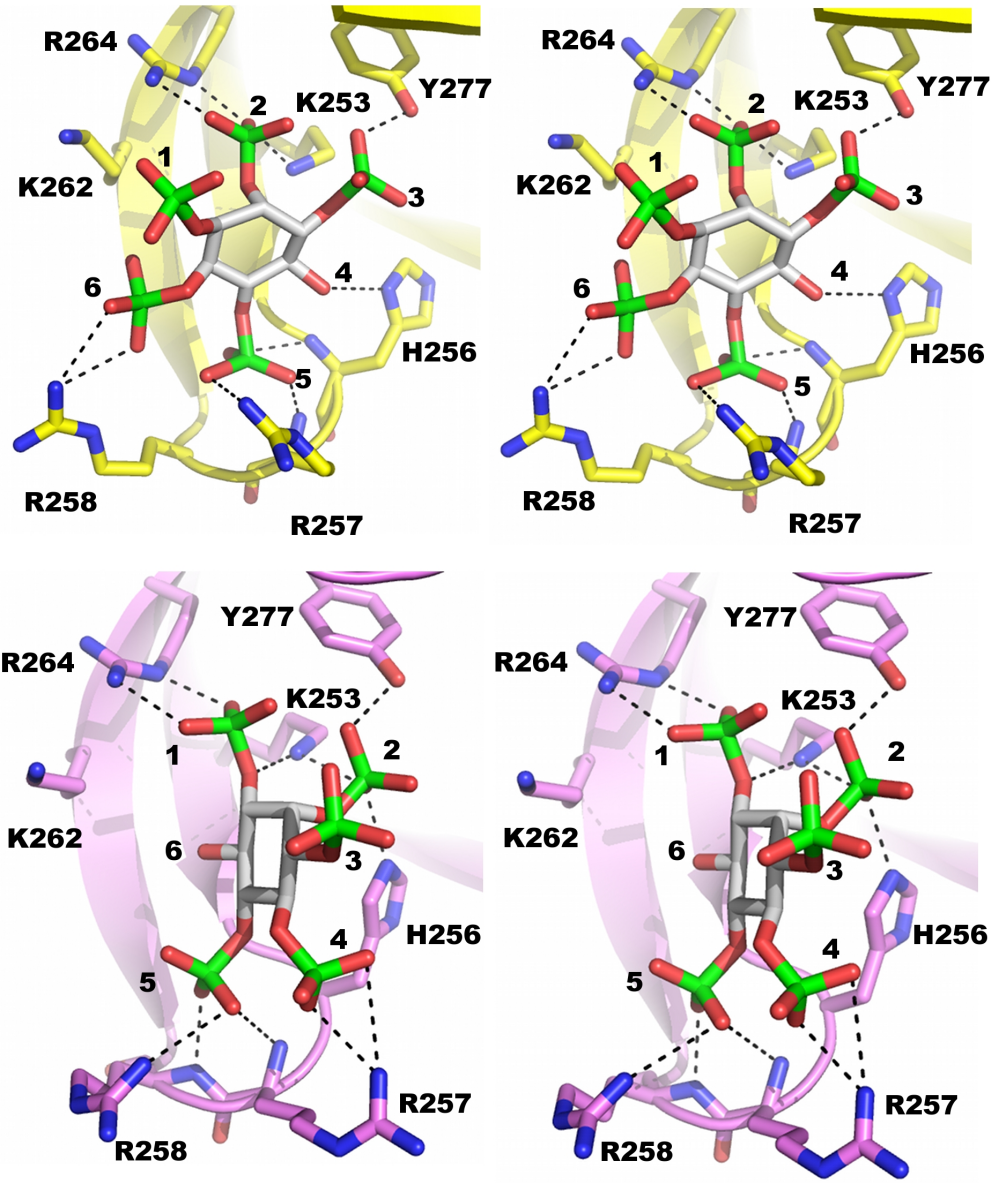
**Specific interactions between CPH and IP_5 _ligands**. Detailed stereo views of the protein/ligand interactions made between CPH and IP_5(4) _and IP_5(6)_. The CPH/IP_5(4) _structure is shown in yellow and the CPH/IP_5(6) _structure in pink. Selected interactions are shown by broken lines.

A closer examination of the ligand binding region for the CPH/IP_5(4) _and CPH/IP_5(6) _structures revealed several important differences. While both IP_5(4) _and IP_5(6) _bind in the same region, each isomer adopts very different orientations in the β1/β2 binding pocket (Figure 4). The inositol rings of both ligands are orientated with the phosphate groups at the 5 position orientated similarly relative to the plane of the β1/β2 loop (Figure 4). The orientation of the two IP_5 _inositol rings differs by a rotation of approximately 90° about the 5 position. This large rotation results in IP_5(6) _making fewer interactions and burying less surface area (387.3 Å^2 ^compared to 431.6 Å^2^) in the binding pocket. These changes provide clear structural evidence for the observed lower binding affinity of IP_5(6) _compared to IP_5(4)_. Interactions formed between the two IP_5 _ligands and CPH are summarized in Table 2. A detailed interaction list can be found in Additional file 1, Table S1. These results demonstrate that a single PH domain can utilize very different modes of interaction and this difference may account for variations in the overall binding of IP isomers. This implies that each IP_5 _could have a special role in differentially regulating PH domain/phosphoinositide signaling.

**Table 2:** CPH/Ligand interaction details.

CPH/IP_5(4)_						CPH/IP_5(6)_					
			Contact						Contact		
Residue	Dist	Surf	H	E	V	Residues	Dist	Surf	H	E	V
K253	2.8	29.7	+	+	+	K253	2.8	46.4	+	+	-
G255	3.0	37.9	+	-	+	G255	3.2	28.2	-	-	+
H256	3.2	33.3	+	-	-	H256	2.8	57.1	+	-	+
R257	2.7	79.2	+	+	-	R257	3.0	62.2	+	+	-
R258	3.1	59.4	+	+	-	R258	2.8	58.6	+	+	-
R259	3.6	2.9	+	+	-	R259					
N260	4.5	9.7	+	-	-	N260					
K262	3.9	39.3	+	+	+	K262	4.9	12.8	+	+	-
R264	2.7	49.7	+	+	-	R264	2.8	47.8	+	+	-
Y277	2.3	52.3	+	-	-	Y277	2.6	41.9	+	-	+
L287	3.8	36.4	+	-	-	L287	3.7	24.8	+	-	-
Y325	5.0	2.8	+	-	-	Y325	3.8	7.4	+	-	+
											
Total		431.6				Total		387.2			

Distances (Dist) are given in angstroms (Å) and represent the distance to the closest protein atom. Surface (Surf) is the contact surface area between the ligand and protein atoms (Å^2^). H - hydrogen bond, E - electrostatic interaction and, V - van der Waals. "+" and "-" indicate observed and not observed interactions respectively.

### Inhibition of PH_Grp1 _and PH_Akt _binding to PtdIns(3,4)P_2 _by IP_5 _isomers

Having shown that CPH possesses a high degree of specificity we next asked whether this property is common to other PH domains. The inhibitory properties of the IP_5 _family were tested against two additional PH domains from Grp1 and Akt. These PH domains have been well characterized structurally making them ideal for comparisons with CPH. The PH domain from Akt is of particular interest since it was recently shown to be regulated by IP_5(2) _interactions [14,19]. The study demonstrated that IP_5(2) _could prevent membrane association of PH_Akt _but did not report the analysis of the other IP_5 _isomers. The results for CPH/IP_5 _binding suggest it is possible that another IP_5 _isomer could be a better inhibitor of PH_Akt _phosphoinositide binding.

**Figure 5:**
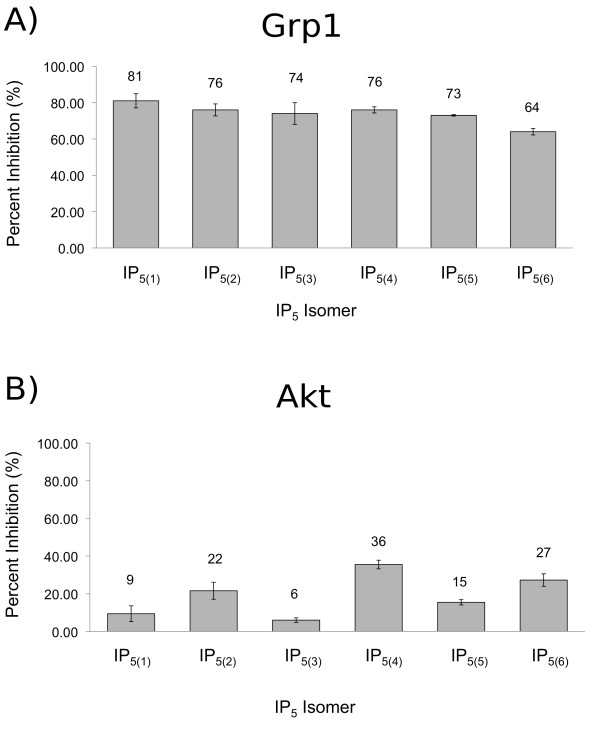
**Inhibition of PH_Grp1 _and PH_Akt _binding**. Inhibition of PH_Grp1 _and PH_Akt _(10 μM) binding to liposomes containing PtdIns(3,4)P_2 _by *myo*-inositol pentakisphosphates (100 μM). Percent inhibition is plotted on the y-axis for the different IP_5 _isomers. Percent inhibition values are shown above the bars. Error bars represent +/- standard error of mean (SEM).

We tested the ability of IP_5 _isomers to inhibit binding of PH_Grp1 _and PH_Akt _to liposomes containing PtdIns(3,4)P_2_. As shown in Figure 5, panel A, PH_Grp1 _binding to PtdIns(3,4)P_2 _was inhibited in a similar way by all IP_5 _isomers (P value 0.1054). Therefore, in contrast to CPH, PH_Grp1 _does not appear to specifically recognize different IP_5 _isomers to any significant extent. Further analysis of the inhibitory properties of these isomers towards Akt/PtdIns(3,4)P_2 _binding did reveal significant differences in specificity, see Figure 5, panel B (P value 0.0002). IP_5(4) _was the most effective inhibitor followed by IP_5(6) _and IP_5(2)_. Weakest inhibition of PH_Akt_/PtdIns(3,4)P_2 _binding was observed for IP_5(3) _followed by IP_5(1) _and IP_5(5) _respectively. These differences suggest that like CPH, PH_Akt _provides sufficient specificity in its binding pocket to differentiate between very similar ligands.

This is a particularly interesting finding given that previous studies have reported that IP_5(2) _is able to prevent Akt localization to membranes by competing with phosphoinositides for binding to its PH domain. As a consequence, the serine phosphorylation and kinase activity of Akt are inhibited resulting in apoptosis in ovarian, lung and breast cancer cells [14,19,20]. Not surprisingly, much interest exists in developing inhibitors to Akt, some of which are based on IP_5(2) _[21,22]. Our results suggest that IP_5(4) _is a more effective inhibitor and may therefore provide a better starting compound for inhibitor design compared to IP_5(2)_.

### Structural determinants of IP_5 _specificity

Collectively these results demonstrate that some PH domains possess enough specificity to differentiate between the six IP_5 _isomers. While varying specificities for different classes of inositol phosphates among PH domains is not a new idea, the ability to recognize very subtle differences, such as those found in the IP_5 _family, has not been well appreciated. In an attempt to understand the structural basis for the high degree of specificity observed we compared the structures of CPH, PH_Grp1 _and PH_Akt_.

**Figure 6:**
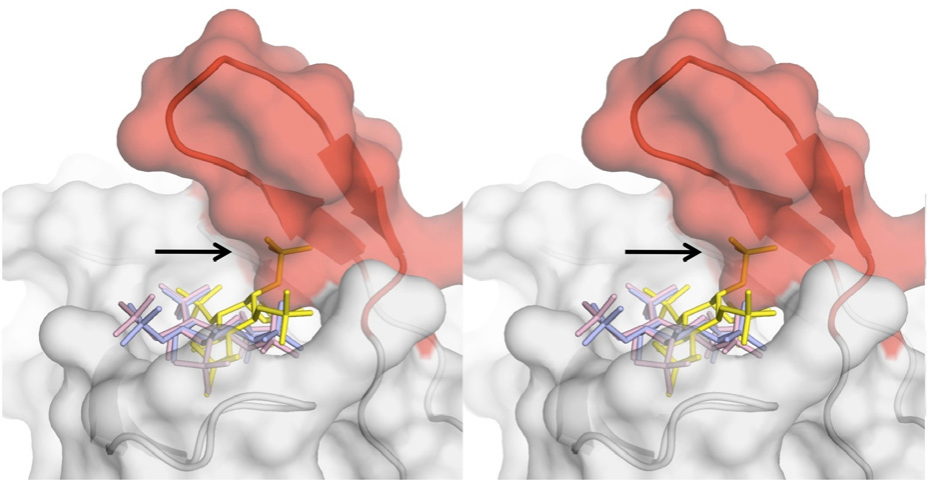
**Surface representation of the PH_Grp1_**. (PDB code: 1FGY) binding pocket shown in stereo. β6/β7 insertion is colored red. IP_5(6) _is colored yellow, IP_5(2) _is colored pink and Ins(1,3,4,5)P_4 _is colored blue.

Examination of the inositol phosphate binding pockets from CPH, PH_Grp1 _and PH_Akt _revealed two important differences. PH_Grp1 _(no IP_5 _specificity) contains 7 basic residues whereas CPH and PH_Akt _(good IP_5 _specificity) contain 6 and 4 basic residues, respectively. This suggests that the number of basic residues in the binding pocket influences IP_5 _specificity. If so, a possible explanation is that too many basic residues physically restrict the possible orientations that IP_5 _isomers can adopt in the binding pocket. Orientation plays an important role in specificity as demonstrated for CPH. Here, IP_5(4) _and IP_5(6) _adopted different orientations in the binding pocket resulting in different binding affinities. It is reasonable to expect that as a binding pocket contains increasing numbers of basic residues the space available for IP_5 _isomers to adopt different orientations will be decreased. The resulting limited orientation(s) may permit a high affinity interaction (due to the abundance of basic residues) but the affinity will be similar for all isomers. In further support of an "orientation-restricted" binding pocket, PH_Grp1 _contains a 20-residue insertion in the β6/β7 loop that forms a β hairpin structure. This β hairpin, not found in CPH or PH_Akt_, folds back and over top of the β1/β2 loop, essentially capping the binding pocket (Figure 6). This added structural feature further restricts possible orientations that could otherwise be adopted by IP_5 _isomers. In support of this, the orientations of IP_5(2) _(PDB code: 1FHW) and that of Ins(1,3,4,5)P_4 _(PDB code: 1FGY) which were both solved bound to Grp1 [3,4], adopt identical orientations in the binding pocket (colored pink and blue respectively in Figure 6). To further illustrate the restricted orientations imposed by these structural features we have aligned the CPH/_IP5(6) _structure with Grp1. The orientation adopted by IP_5(6) _is forbidden in the binding pocket of Grp1 due to steric clashes with the β6/β7 insertion. It is also possible that since basic residues have long and flexible side chains, PH domains with a greater number of basic residues are more adaptable to different ligands and can therefore accommodate different inositol phosphate molecules with similar affinities.

## Conclusion

Work presented here substantiates the growing body of evidence that IP_5_'s regulate PH domain/phosphoinositide interactions [14,17,19,20]. Specifically, these results demonstrate that the six IP_5 _isomers differ in their inhibitory properties towards PH domains. This allows for the potential to fine tune PH domain/phosphoinositide signaling pathways. Our work has direct implications for Akt signaling as previous studies have shown that IP_5(2) _can inhibit membrane binding and subsequent activation of Akt [14,19,20]. In light of our findings, it would be prudent to include all IP_5 _isomers in future binding studies involving PH domains and IP_5_. The potential for a high degree of specificity demonstrated here could in theory be extended to other families of inositol phosphates. Given that there are 63 possible inositol phosphates this provides nature with an expansive toolbox for regulating phosphoinositide based signaling pathways.

In addition to their potential role as signaling molecules, IP_5_'s could also be used as inhibitors to probe biological pathways. A potential issue in administering exogenous IP_5_'s is their low cell permeability. Fortunately, much progress has been made towards overcoming this problem. By masking phosphate and hydroxyl groups as esters, the hydrophobicity and membrane permeability is significantly increased [23]. Once inside the cell the ester groups are removed by host esterases yielding the original IP_5 _molecule (available from SiChem). A similar approach can now be applied to phosphoinositides yielding new cell permeant tools for studying these signaling pathways [24,25].

## Methods

### Protein expression and purification

CPH was expressed and purified as described previously [17]. PH_Grp1 _(residues 260-390) was cloned into the pDEST17 expression vector (Invitrogen). *Escherichia coli *BL21(DE3) cells were grown in standard LB medium supplemented with 10 mg ml^-1 ^ampicillin at 37.0°C with shaking (225 rev min^-1^) until the light absorbance at 600 nm reached 0.5. The temperature was then lowered to 20.0°C and protein expression was induced using 1.0 mM IPTG. Following a 5 hour induction period cells were harvested by centrifugation at 3 315 × G and 4.0°C for 15 minutes. The resulting cell pellets were flash frozen in liquid nitrogen and stored at -80.0°C. Prior to cell lysis using a French press, pellets were resuspended in 35 ml with NiA buffer (20 mM Tris-HCl pH 7.5, 1 M KCl, 5 mM imidazole and 10% glycerol). Cell lysates were centrifuged at 48 384 × G and 4.0°C for 45 minutes. The resulting supernatant was applied to a HiTrap Nickel affinity column (GE Healthcare). PH_Grp1 _was eluted from the column using NiA buffer supplemented with 500 mM imidazole following sequential washes with NiA buffer containing 20 and 45 mM imidazole. The protein sample was buffer exchanged into 20 mM Tris-HCl pH 7.5 and 300 mM KCl using a HiPrep 26/10 desalting column (GE Healthcare). The hexahistidine tag was removed by cleavage with TEV protease. This resulted in four residues (Gly, Ser, Phe and Thr) being retained on the N-terminal side of the first residue of PH_Grp1_. The salt concentration was diluted to 100 mM KCl using SA buffer (20 mM Tris-HCl pH 7.5) and the sample applied to a HiTrap SP Sepharose ion exchange column (GE Healthcare). PH_Grp1 _was eluted using an increasing salt gradient through the application of increasing amounts of SB buffer (20 mM Tris-HCl pH 7.5 and 1 M KCl). The resulting PH_Grp1 _sample was buffer exchanged into 20 mM Tris-HCl pH 7.5, 100 mM KCl, 2 mM TCEP and 10% glycerol and subsequently concentrated using a centrifugal filter. PH_Grp1 _purified in this manner is greater the 95% pure as judged by SDS-PAGE. PH_Akt _(residues 1-123) was cloned into the pDEST-HisMBP expression vector (Addgene plasmid #11085). The fusion protein was expressed and purified in the same manner as PH_Grp1_.

### Crystallization and data collection of the CPH/IP_5(6) _complex

CPH (2.5 mg ml^-1^) was crystallized in the presence of 1 mM IP_5(6) _(Sichem GmbH, Germany) using the hanging drop vapour diffusion method under the following conditions. Inositol phosphates are purified by HPLC to greater the 98% purity. A 3 μl drop containing 2 μl of CPH (2.5 mg ml^-1^) and 1 mM IP_5(6) _in the crystallization buffer described above and 1 μl of 0.1 M Bis-Tris pH 6.5 and 28% polyethylene glycol 2000 monomethyl ether was suspended over 500 μl of 0.45 M ammonium sulfate and incubated at 21.0°C. Crystals possessing bypyramidal morphology grew to their maximum size after 72 hours. A single high-resolution data set (1.7 Å) was collected at a wavelength of 0.9797 Å at beamline X26-C of the Brookhaven National Laboratory using a ADSC Quantum-4 CCD area detector. The data were processed using the HKL2000 program suite [26].

### CPH/IP_5(6) _structure determination and model refinement

The crystal structure of CPH in complex with IP_5(6) _was solved by molecular replacement using the program *MOLREP *[27]. The search model used in molecular replacement was the crystal structure of apo-CPH (PDB code 1ZM0). Iterative cycles of model building and refinement were carried out using the programs *WinCoot *[28] and *Refmac5 *[29] respectively. Ligand-Protein Contacts (LPC) were derived with LPC software [30]. All figures describing protein structures presented in this report were generated using *PyMol *[31].

### PH domain/IP_5 _inhibition assays

All measurements were made using a ProteOn XPR36 surface plasmon resonance instrument equipped with an NLC sensor chip (Biorad). The sensor chip surface was pre-treated with three sequential injections (30 μl min^-1 ^for 1 min) of SPR buffer (20 mM Tris-HCl pH 7.5, 100 mM KCl and 2% glycerol), 5 mM NaOH and SPR buffer. Liposomes containing 5 mole % PtdIns(3,4)P_2 _and 1 mole % biotinylated phosphatidylethanolamine (Echelon Biosciences) were diluted to 10 μM in SPR buffer and applied in sequential injections (25 μl min^-1 ^for 3 mins) until approximately 1000 response units (RU) had been applied to the sensor chip surface. The liposome surface was then washed with a single injection (30 μl min^-1 ^for 1 min) of 1 mM NaOH. PH domains (final concentration of CPH was 50 μM and was 10 μM for PH_Grp1 _and PH_Akt_), diluted in SPR buffer, were injected (30 μl min^-1 ^for 2 mins) to determine the level of binding in the absence of inhibitor. For inhibition experiments, PH domains were incubated with specific IP_5 _isomers prior to injection across the liposome surface. PH domain/IP_5 _samples were injected at 30 μl/min for 2 minutes. Surfaces were regenerated with 2 sequential injections of 5 mM NaOH at 50 μl min^-1 ^for 30 seconds. Sample signals were corrected by subtracting the signal obtained by injecting sample across liposome free surfaces. The PH domain/IP_5 _signals were compared to PH domain only signals to determine the relative inhibitory properties of each IP_5 _isomer. This comparison was made after binding of PH domain and PH domain/IP_5 _samples reached steady state equilibrium. To calculate percent inhibition, the signal from a specific time point, when binding had reached equilibrium, was taken for all samples. The signal for PH domain alone samples was used as the standard for complete binding (100%). Signal obtained for PH domain/IP_5 _samples were compared to this standard to determine the percent inhibition. All measurements were made in triplicate. All experiments were conducted at 21.0°C. The statistical significance of differences in the inhibitory properties was assessed by an analysis of variance using the GraphPad InStat software package (GraphPad Software).

## Abbreviations

PH domain: pleckstrin homology domain; IP: inositol phosphate; PtdIns: phosphoinositide; IP_3_: *myo*-inositol-1:4,5-trisphosphate; PtdIns(4, 5)P_2_: phosphatidylinositol 4, 5-bisphosphate; PtdIns(3,4,5)P_3_: phosphatidylinositol 3, 4, 5-trisphosphate; PtdIns(3, 4)P_2_: phosphatidylinositol 3, 4-bisphosphate; Akt: protein kinase B; IP_5(#)_: *myo*-inositol pentakisphosphate, number in brackets indicates position missing a phosphate; CPH: carboxy terminal PH domain of pleckstrin; Grp1: general receptor for phosphoinositides isoform 1; PH_Akt_: PH domain from Akt; PH_Grp1_: PH domain from Grp1.

## Authors' contributions

SGJ designed experiments, purified CPH, PH_Grp1 _and PH_Akt_, crystallized the complex, collected and processed crystallographic data, refined the structure, conducted binding assay and prepared the manuscript. SAS purified CPH, PH_Grp1 _and PH_Akt_, conducted binding assays and prepared the manuscript. CS designed experiments and wrote the manuscript. MSJ designed and oversaw the experiments and wrote the manuscript. All authors have read and approved the manuscript.

## Supplementary Material

Additional File 1 **Interactions between CPH and IP_5 _ligands**. This file provides a table listing all of the interactions and distances observed between CPH and IP_5(4) _and IP_5(6) _isomers. Click here for file

## References

[B1] Letunic I Copley RR Pils B Pinkert S Schultz J Bork P SMART 5: domains in the context of genomes and networks Nucleic Acids Res 2006 34 Database D257 60 10.1093/nar/gkj079 16381859 PMC1347442

[B2] Cozier GE Carlton J Bouyoucef D Cullen PJ Membrane targeting by pleckstrin homology domains Curr Top Microbiol Immunol 2004 282 49 88 14594214 10.1007/978-3-642-18805-3_3

[B3] Lietzke SE Bose S Cronin T Klarlund J Chawla A Czech P Lambright DG Structural basis of 3-phosphoinositide recognition by pleckstrin homology domains Mol Cell 2000 6 2 385 10.1016/S1097-2765(00)00038-1 10983985

[B4] Ferguson KM Kavran JM Sankaran VG Fournier E Isakoff SJ Skolnik EY Lemmon MA Structural basis for discrimination of 3-phosphoinositides by pleckstrin homology domains Mol Cell 2000 6 2 373 384 10.1016/S1097-2765(00)00037-X 10983984

[B5] Harlan JE Hajduk PJ Yoon HS Fesik SW Pleckstrin homology domains bind to phosphatidylinositol-4,5-bisphosphate Nature 1994 371 6493 168 170 10.1038/371168a0 8072546

[B6] Harlan JE Yoon HS Hajduk PJ Fesik SW Structural characterization of the interaction between a pleckstrin homology domain and phosphatidylinositol 4,5-bisphosphate Biochemistry 1995 34 31 9859 9864 10.1021/bi00031a006 7632686

[B7] Balla T Szentpetery Y Kim J Phosphoinositide Signaling: New Tools and Insights Physiology 2009 24 4 231 10.1152/physiol.00014.2009 19675354 PMC3126675

[B8] Irvine RF Schell MJ Back in the water: the return of the inositol phosphates Nat Rev Mol Cell Biol 2001 2 5 327 338 10.1038/35073015 11331907

[B9] York JD Guo S Odom AR Spiegelberg BD Stolz LE An expanded view of inositol signaling Adv Enzyme Regul 2001 41 57 71 10.1016/S0065-2571(00)00025-X 11384737

[B10] Downes CP Inositol phosphates: a family of signal molecules? Trends Neurosci 1988 11 8 336 10.1016/0166-2236(88)90051-3 2469189

[B11] Downes CP Macphee CH myo-inositol metabolites as cellular signals Eur J Biochem 1990 193 1 10.1111/j.1432-1033.1990.tb19297.x 2171926

[B12] Berridge MJ Irvine RF Inositol phosphates and cell signalling Nature 1989 341 6239 197 205 10.1038/341197a0 2550825

[B13] Kavran JM Klein DE Lee A Falasca M Isakoff SJ Skyolnik EY Lemmon MA Specificity and promiscuity in phosphoinositide binding by pleckstrin homology domains J Biol Chem 1998 273 30497 9804818 10.1074/jbc.273.46.30497

[B14] Piccolo E Vignati S Maffucci T Innominato PF Riley AM Potter BV Pandolfi PP Broggini M Iacobelli S Innocenti P Falasca M Inositol pentakisphosphate promotes apoptosis through the PI 3-K/Akt pathway Oncogene 2004 23 9 1754 1765 10.1038/sj.onc.1207296 14755253

[B15] Hirose K Kadowaki S Tanabe M Takeshima H Iino M Spatiotemporal dynamics of inositol 1,4,5-trisphosphate that underlies complex Ca^2+ ^mobilization patterns Science 1999 283 1527 10.1126/science.284.5419.1527 10348740

[B16] Edlich C Stier G Simon B Sattler M Muhle-Goll C Structure and phosphatidylinositol-(3,4)-bisphosphate binding of the C-terminal PH domain of human pleckstrin Structure 2005 13 2 277 286 10.1016/j.str.2004.11.012 15698571

[B17] Jackson SG Zhang Y Haslam RJ Junop MS Structural analysis of the carboxy terminal PH domain of pleckstrin bound to D-myo-inositol 1,2,3,5,6-pentakisphosphate BMC Structural Biology 2007 7 80 18034889 10.1186/1472-6807-7-80 PMC2200656

[B18] Jackson SG Zhang Y Bao X Zhang K Summerfield R Haslam RJ Junop MS Structure of the carboxy-terminal PH domain of pleckstrin at 2.1 Angstroms Acta Crystallogr D Biol Crystallogr 2006 62 Pt 3 324 330 10.1107/S0907444905043179 16510979

[B19] Maffucci T Piccolo E Cumashi A Iezzi M Riley AM Saiardi A Godage HY Rossi C Broggini M Iacobelli S Potter BV Innocenti P Falasca M Inhibition of the phosphatidylinositol 3-kinase/Akt pathway by inositol pentakisphosphate results in antiangiogenic and antitumor effects Cancer Res 2005 65 18 8339 8349 10.1158/0008-5472.CAN-05-0121 16166311

[B20] Razzini G Berrie CP Vignati S Broggini M Mascetta G Brancaccio A Falasca M Novel functional PI 3-kinase antagonists inhibit cell growth and tumorigenicity in human cancer cell lines FASEB Journal 2000 14 9 1179 10834940 10.1096/fasebj.14.9.1179

[B21] Lindsley CW Stanley BF Yaroschak M Bilodeau MT Layton ME Recent progress in the development of ATP-competitive and allosteric Akt kinase inhibitors Curr Top Med Chem 2007 7 14 1349 10.2174/156802607781696864 17692025

[B22] Lindsley CW Stanley BF Layton ME Bilodeau MT The PI3K/Akt pathways: recent progress in the development of ATP-competitive and allosteric Akt kinase inhibitors Curr Cancer Drug Targets 2008 8 1 7 10.2174/156800908783497096 18288939

[B23] Schultz C Prodrugs of biologically active phosphate esters Bioorg Med Chem 2003 11 6 885 10.1016/S0968-0896(02)00552-7 12614874

[B24] Subramanian D Laketa V Muller R Tischer C Zarbakhsh S Pepperkok R Schultz C Activation of membrane-permeant caged PtdIns(3)P induces endosomal fusion in cells Nat Chem Biol 2010 6 5 324 10.1038/nchembio.348 20364126

[B25] Laketa V Zarbakhsh S Morbier E Subramanian D Dinkel C Brumbaugh J Zimmermann P Pepperkok R Schultz C Membrane-Permeant Phosphoinositide Derivatives as Modulators of Growth Factor Signaling and Neurite Outgrowth Chem and Biol 2009 16 1190 10.1016/j.chembiol.2009.10.005 19942142

[B26] Otwinowski Z Minor W Processing of X-ray Diffraction Data Collected in Oscillation Mode Methods in Enzymology 1997 276 Macromolecular Crystallography, part A 307 326 full_text 10.1016/S0076-6879(97)76066-X 27754618

[B27] Vagin A Teplyakov A MOLREP: an automated program for molecular replacement J Appl Cryst 1997 30 1022 1025 10.1107/S0021889897006766

[B28] Emsley P Cowtan K Coot: model-building tools for molecular graphics Acta Crystallogr D Biol Crystallogr 2004 60 Pt 12 Pt 1 2126 2132 10.1107/S0907444904019158 15572765

[B29] Murshudov GN Vagin AA Dodson EJ Refinement of macromolecular structures by the maximum-likelihood method Acta Crystallogr D Biol Crystallogr 1997 53 Pt 3 240 255 10.1107/S0907444996012255 15299926

[B30] Sobolev V Sorokine A Prilusky J Abola EE Edelman M Automated analysis of interatomic contacts in proteins Bioinformatics 1999 15 327 10.1093/bioinformatics/15.4.327 10320401

[B31] The PyMOL Molecular Graphics System http://www.pymol.org

