# Supplementary material for: Inositol pentakisphosphate isomers bind PH domains with varying specificity and inhibit phosphoinositide interactions
Source: BMC Struct Biol. 2011 Feb 10;11:11. doi: 10.1186/1472-6807-11-11 (PMC3042905; doi:10.1186/1472-6807-11-11)
Supplement: Additional File 1 — Interactions between CPH and IP5 ligands. This file provides a table listing all of the interactions and distances observed between CPH and IP5(4) and IP5(6) isomers. [file 1472-6807-11-11-S1.DOC]

| **CPH/IP_5(4)_** | | | | |  | **CPH/IP_5(6)_** | | | | |  |  |  |  |
| --- | --- | --- | --- | --- | --- | --- | --- | --- | --- | --- | --- | --- | --- | --- |
| **Ligand** |  | **Protein** | | |  | **Ligand** |  | **Protein** | | |  |  |  |  |
|  |  | **Residue** | **Atom** | **Dist.** |  |  |  | **Residue** | **Atom** | **Dist.** |  |  |  |  |
| **PO_4_(1)** |  |  |  |  |  | **PO_4_(1)** |  |  |  |  |  |  |  |  |
| C1 |  | K262 | CD | 5.2 |  | O11 |  | K253 | NZ | 3.1 |  |  |  |  |
| O31 |  | L287 | CD2 | 5.8 |  | O21 |  | R264 | NH2 | 2.9 |  |  |  |  |
| O31 |  | R264 | NH2 | 4.5 |  | O21 |  | R264 | NE | 3.7 |  |  |  |  |
| O31 |  | K262 | CD | 4.3 |  | O21 |  | K262 | NZ | 4.7 |  |  |  |  |
| O41 |  | L287 | CD2 | 5.6 |  | O21 |  | K262 | CD | 4.9 |  |  |  |  |
|  |  |  |  |  |  | O21 |  | K262 | CE | 5.5 |  |  |  |  |
|  |  |  |  |  |  | O21 |  | L287 | CD2 | 5.1 |  |  |  |  |
|  |  |  |  |  |  | O31 |  | R264 | NE | 2.8 |  |  |  |  |
|  |  |  |  |  |  | O31 |  | K253 | NZ | 2.8 |  |  |  |  |
|  |  |  |  |  |  | O31 |  | R264 | CD | 3.6 |  |  |  |  |
|  |  |  |  |  |  | O31 |  | Y277 | CE1 | 3.6 |  |  |  |  |
|  |  |  |  |  |  | O31 |  | Y277 | CD1 | 3.9 |  |  |  |  |
|  |  |  |  |  |  | O31 |  | L287 | CD1 | 4.0 |  |  |  |  |
|  |  |  |  |  |  | O41 |  | Y277 | CE1 | 3.5 |  |  |  |  |
|  |  |  |  |  |  | O41 |  | L287 | CB | 3.7 |  |  |  |  |
|  |  |  |  |  |  | O41 |  | L287 | O | 4.1 |  |  |  |  |
|  |  |  |  |  |  | O41 |  | L287 | CD2 | 4.9 |  |  |  |  |
|  |  |  |  |  |  |  |  |  |  |  |  |  |  |  |
| **PO_4_(2)** |  |  |  |  |  | **PO_4_(2)** |  |  |  |  |  |  |  |  |
| O12 |  | K262 | CD | 5.1 |  | O12 |  | H256 | CD2 | 3.6 |  |  |  |  |
| O12 |  | K253 | NZ | 3.5 |  | O22 |  | Y277 | OH | 2.6 |  |  |  |  |
| O22 |  | R264 | NH2 | 2.7 |  | O22 |  | Y277 | CZ | 3.3 |  |  |  |  |
| O22 |  | L287 | CD1 | 3.8 |  | O22 |  | Y277 | CE1 | 3.4 |  |  |  |  |
| O22 |  | L287 | CD2 | 3.9 |  | O22 |  | L287 | O | 4.2 |  |  |  |  |
| O32 |  | L287 | CB | 3.9 |  | O32 |  | Y277 | OH | 3.7 |  |  |  |  |
| O32 |  | L287 | CD2 | 4.2 |  | O32 |  | H256 | NE2 | 3.8 |  |  |  |  |
| O32 |  | L287 | O | 4.4 |  | O32 |  | H256 | CD2 | 4.3 |  |  |  |  |
| O42 |  | R264 | NE | 2.7 |  | O32 |  | Y325 | OH | 4.6 |  |  |  |  |
| O42 |  | R264 | NE | 2.7 |  | O42 |  | H256 | NE2 | 2.8 |  |  |  |  |
| O42 |  | K253 | NZ | 2.8 |  | O42 |  | K253 | NZ | 2.9 |  |  |  |  |
| O42 |  | R264 | CD | 3.6 |  | O42 |  | Y277 | OH | 3.4 |  |  |  |  |
| O42 |  | K253 | CE | 3.9 |  | O42 |  | K253 | CE | 3.5 |  |  |  |  |
| O42 |  | Y277 | CE1 | 4.1 |  | O42 |  | H256 | CE1 | 3.8 |  |  |  |  |
| O42 |  | L287 | CD1 | 4.1 |  | O42 |  | Y325 | CE2 | 3.8 |  |  |  |  |
| 042 |  | K262 | CD | 4.9 |  |  |  |  |  |  |  |  |  |  |
|  |  |  |  |  |  |  |  |  |  |  |  |  |  |  |
| **PO_4_(3)** |  |  |  |  |  | **PO_4_(3)** |  |  |  |  |  |  |  |  |
| O13 |  | K253 | NZ | 3.4 |  |  |  |  |  |  |  |  |  |  |
| O13 |  | H256 | ND1 | 4.6 |  |  |  |  |  |  |  |  |  |  |
| O23 |  | Y277 | OH | 3.3 |  |  |  |  |  |  |  |  |  |  |
| O23 |  | H256 | ND1 | 4.4 |  |  |  |  |  |  |  |  |  |  |
| O23 |  | H256 | CE1 | 4.5 |  |  |  |  |  |  |  |  |  |  |
| O23 |  | K253 | CE | 4.7 |  |  |  |  |  |  |  |  |  |  |
| O23 |  | Y325 | CE2 | 5.0 |  |  |  |  |  |  |  |  |  |  |
| O23 |  | Y325 | OH | 5.3 |  |  |  |  |  |  |  |  |  |  |
| O33 |  | L287 | O | 4.5 |  |  |  |  |  |  |  |  |  |  |
| O43 |  | Y277 | OH | 2.3 |  |  |  |  |  |  |  |  |  |  |
| O43 |  | Y277 | CE1 | 2.8 |  |  |  |  |  |  |  |  |  |  |
| O43 |  | Y277 | CZ | 2.9 |  |  |  |  |  |  |  |  |  |  |
| O43 |  | K253 | NZ | 3.8 |  |  |  |  |  |  |  |  |  |  |
| O43 |  | L287 | O | 4.1 |  |  |  |  |  |  |  |  |  |  |
|  |  |  |  |  |  |  |  |  |  |  |  |  |  |  |
| **PO_4_(4)** |  |  |  |  |  | **PO_4_(4)** |  |  |  |  |  |  |  |  |
| C4 |  | G255 | CA | 3.9 |  | C1 |  | H256 | CD2 | 4.2 |  |  |  |  |
| C4 |  | K253 | NZ | 4.1 |  | O14 |  | R258 | NH1 | 5.4 |  |  |  |  |
| O14 |  | H256 | N | 3.4 |  | O24 |  | R257 | NH1 | 3.6 |  |  |  |  |
| O14 |  | H256 | ND1 | 3.5 |  | O24 |  | R257 | CZ | 4.5 |  |  |  |  |
| O14 |  | H256 | CB | 4.1 |  | O24 |  | R257 | CD | 4.5 |  |  |  |  |
|  |  |  |  |  |  | O24 |  | R258 | NH1 | 5.3 |  |  |  |  |
|  |  |  |  |  |  | O34 |  | R257 | NH1 | 3.4 |  |  |  |  |
|  |  |  |  |  |  | O34 |  | H256 | CD2 | 4.7 |  |  |  |  |
|  |  |  |  |  |  | O44 |  | R257 | NH1 | 3.0 |  |  |  |  |
|  |  |  |  |  |  | O44 |  | R257 | CD | 3.4 |  |  |  |  |
|  |  |  |  |  |  | O44 |  | R257 | CG | 3.6 |  |  |  |  |
|  |  |  |  |  |  | O44 |  | R257 | N | 4.2 |  |  |  |  |
|  |  |  |  |  |  | O44 |  | H256 | CB | 4.5 |  |  |  |  |
|  |  |  |  |  |  |  |  |  |  |  |  |  |  |  |
| **PO_4_(5)** |  |  |  |  |  | **PO_4_(5)** |  |  |  |  |  |  |  |  |
| C5 |  | G255 | CA | 4.1 |  | O15 |  | H256 | N | 3.3 |  |  |  |  |
| C5 |  | R257 | NH2 | 5.0 |  | O25 |  | R258 | NH1 | 3.5 |  |  |  |  |
| O15 |  | G255 | CA | 3.3 |  | O25 |  | R258 | CD | 4.5 |  |  |  |  |
| O25 |  | R257 | NH2 | 3.1 |  | O25 |  | R258 | CB | 4.5 |  |  |  |  |
| O25 |  | R257 | NE | 3.6 |  | O35 |  | R258 | N | 2.8 |  |  |  |  |
| O25 |  | R257 | CB | 4.0 |  | O35 |  | R257 | N | 3.0 |  |  |  |  |
| O25 |  | R258 | CG | 4.1 |  | O35 |  | H256 | N | 3.1 |  |  |  |  |
| O25 |  | R258 | NE | 4.2 |  | O35 |  | R258 | CB | 3.5 |  |  |  |  |
| O35 |  | G255 | CA | 3.0 |  | O35 |  | G255 | CA | 3.2 |  |  |  |  |
| O35 |  | G255 | C | 3.0 |  | O45 |  | R257 | CB | 3.2 |  |  |  |  |
| O35 |  | R258 | N | 3.2 |  | O45 |  | R257 | N | 3.4 |  |  |  |  |
| O35 |  | G255 | O | 3.5 |  | O45 |  | R258 | NH1 | 3.5 |  |  |  |  |
| O35 |  | K259 | N | 3.6 |  |  |  |  |  |  |  |  |  |  |
| O35 |  | R258 | CA | 4.0 |  |  |  |  |  |  |  |  |  |  |
| O35 |  | R258 | CB | 4.1 |  |  |  |  |  |  |  |  |  |  |
| O35 |  | N260 | N | 4.5 |  |  |  |  |  |  |  |  |  |  |
| O35 |  | N260 | CB | 5.6 |  |  |  |  |  |  |  |  |  |  |
| O45 |  | R257 | N | 2.7 |  |  |  |  |  |  |  |  |  |  |
| O45 |  | R257 | CB | 2.7 |  |  |  |  |  |  |  |  |  |  |
| O45 |  | R257 | NE | 3.0 |  |  |  |  |  |  |  |  |  |  |
| O45 |  | H256 | N | 3.2 |  |  |  |  |  |  |  |  |  |  |
| O45 |  | R257 | CG | 3.3 |  |  |  |  |  |  |  |  |  |  |
| O45 |  | R257 | CD | 3.7 |  |  |  |  |  |  |  |  |  |  |
| O45 |  | H256 | CA | 3.7 |  |  |  |  |  |  |  |  |  |  |
| O45 |  | H256 | CB | 4.0 |  |  |  |  |  |  |  |  |  |  |
|  |  |  |  |  |  |  |  |  |  |  |  |  |  |  |
| **PO_4_(6)** |  |  |  |  |  | **PO_4_(6)** |  |  |  |  |  |  |  |  |
| C6 |  | G255 | CA | 5.1 |  | C6 |  | G255 | CA | 4.2 |  |  |  |  |
| C6 |  | K262 | CD | 5.3 |  | C6 |  | H256 | CD2 | 4.3 |  |  |  |  |
| O16 |  | R257 | NH2 | 4.4 |  | C6 |  | K253 | NZ | 4.6 |  |  |  |  |
| O26 |  | R258 | NH2 | 3.1 |  | O16 |  | G255 | CA | 3.6 |  |  |  |  |
| O26 |  | R258 | NE | 3.2 |  | O16 |  | K253 | NZ | 4.7 |  |  |  |  |
| O26 |  | R258 | CZ | 3.7 |  | O16 |  | K262 | CD | 5.1 |  |  |  |  |
| O26 |  | R258 | CB | 4.4 |  | O16 |  | K262 | CE | 5.4 |  |  |  |  |
| O26 |  | R257 | NH2 | 4.9 |  | O16 |  | K262 | CG | 5.4 |  |  |  |  |
| O26 |  | N260 | ND2 | 4.9 |  |  |  |  |  |  |  |  |  |  |
| O26 |  | N260 | CB | 5.7 |  |  |  |  |  |  |  |  |  |  |
| O36 |  | K262 | CD | 3.9 |  |  |  |  |  |  |  |  |  |  |
| O36 |  | K262 | NZ | 4.7 |  |  |  |  |  |  |  |  |  |  |
| O36 |  | K262 | CG | 4.8 |  |  |  |  |  |  |  |  |  |  |
| O36 |  | R258 | NH2 | 4.9 |  |  |  |  |  |  |  |  |  |  |
| O36 |  | N260 | CB | 5.8 |  |  |  |  |  |  |  |  |  |  |
| O46 |  | R258 | NH2 | 3.3 |  |  |  |  |  |  |  |  |  |  |
| O46 |  | R257 | NH2 | 5.3 |  |  |  |  |  |  |  |  |  |  |

Distances (Dist) are given in angstroms, Å. Ligand atoms and protein residues are numbered according to the PDB file, 2I5C.
